# Recent Developments in Tough Hydrogels for Biomedical Applications

**DOI:** 10.3390/gels4020046

**Published:** 2018-05-22

**Authors:** Yuan Liu, Weilue He, Zhongtian Zhang, Bruce P. Lee

**Affiliations:** 1 Department of Chemical Engineering, University of Massachusetts Amherst, Amherst, MA 01003, USA; yuanl@umass.edu; 2 FM Wound Care LLC, Hancock, MI 49930, USA; weiluehe@fmwoundcare.com; 3 Department of Biomedical Engineering, Michigan Technological University, Houghton, MI 49931, USA; zzhang11@mtu.edu

**Keywords:** tough hydrogels, biomedical applications, tissue adhesives, tissue engineering, soft actuators

## Abstract

A hydrogel is a three-dimensional polymer network with high water content and has been attractive for many biomedical applications due to its excellent biocompatibility. However, classic hydrogels are mechanically weak and unsuitable for most physiological load-bearing situations. Thus, the development of tough hydrogels used in the biomedical field becomes critical. This work reviews various strategies to fabricate tough hydrogels with the introduction of non-covalent bonds and the construction of stretchable polymer networks and interpenetrated networks, such as the so-called double-network hydrogel. Additionally, the design of tough hydrogels for tissue adhesive, tissue engineering, and soft actuators is reviewed.

## 1. Introduction

Hydrogels are an important class of biomaterial, consisting of a three-dimensional (3D) polymer network with water content as high as over 99.9 wt %. Hydrogels have been utilized in various biomedical applications, functioning as tissue adhesives [1,2], tissue engineering scaffolds [3,4], drug delivery carriers [5,6], biosensors [7,8], and soft robotic and electronic components [9,10,11,12]. Properties of these materials are easily controlled by their preparation method and composition, so that hydrogels can be created with desired mechanical properties, biocompatibility, biodegradability, permeability, and responsivity to environmental cues [13,14].

**Figure 1:**
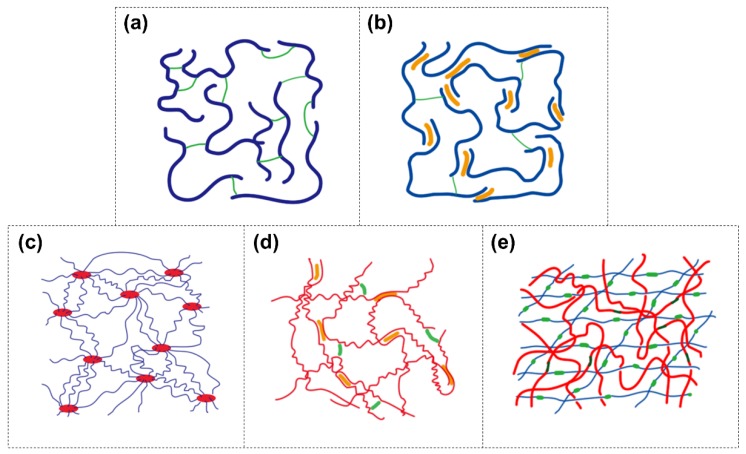
Schematic of (**a**) the classic covalent single-network hydrogel, (**b**) dual-crosslinked hydrogel, (**c**) polymer-intercalated nanocomposite hydrogel, (**d**) hydrogel with elastomer-like segments and non-covalent bonds, and (**e**) double-network (DN) hydrogel. Blue and red long lines: polymer backbones; short green lines: covalent crosslinking points; yellow short lines: non-covalent crosslinking points.

Hydrogels resemble the extracellular matrix (ECM) in biological tissues, which is mainly composed of macromolecular components, such as fibrous collagens and proteoglycans. However, unlike naturally-occurring ECM that can withstand large deformations and loads, classic covalent single network hydrogels (Figure 1a) are mechanically weak [15], which makes them unsuitable for most physiological load-bearing situations. Many approaches have been used to improve the toughness of hydrogels, for example, non-covalent bonds (e.g., hydrogen bonds [16] and ionic interactions [17]) toughened hydrogels, hydrogels with highly stretchable networks [18,19], and double-network (DN) hydrogels [20]. Herein we introduce the strategies to construct tough hydrogels using these approaches. Recently-reported tough hydrogels for biomedical applications are reviewed. We firstly introduce the tough hydrogels used for tissue adhesives. Biomimetic adhesives, nanocomposite adhesives, and interpenetrating network (IPN) hydrogels as adhesives are covered. Then we focus on the tough hydrogels for tissue engineering, highlighting the improvement of hydrogel biocompatibility and mechanical properties in acellular and cell-laden hydrogel scaffold fabrication and 3D printing. Tough hydrogels used for repairing load-bearing soft tissues (i.e., cartilage, cornea, and cardiovascular tissue) are illustrated. Finally, we review tough hydrogel-based actuators that can respond to various stimuli and perform repeated bending movements. Both homogeneous and bi-layered designs are introduced.

## 2. Strategies to Construct Tough Hydrogels

Incorporating mechanisms to dissipate fracture energy into the network is critical to design a tough hydrogel [15]. In general, the dissipation of fracture energy can fall into two categories: (1) breakage of the sacrificial bonds and (2) stretching of the polymer network (viscous dissipation). In this section, multiple strategies to construct tough hydrogels are reviewed.

### 2.1. Covalent Network with Non-Covalent Crosslinks (Dual-Crosslink)

Dual-crosslinked hydrogels contain both covalent and non-covalent bonds in the network (Figure 1b). Usually the covalent bonds possess higher binding energies than the non-covalent bonds (e.g., hydrogen bonds, hydrophobic associations, ionic interactions, dipole-dipole interactions, ion-dipole interactions, cation–π interactions, host-guest interactions, π–π stacking, and polymer-nanoparticle interfacial bonds) [21]. When the dual-crosslinked hydrogels deform, the weaker non-covalent bonds break to dissipate energy as sacrificial bonds, while the covalent bonds are preserved. Incorporation of one type (e.g., hydrogen bonds [22,23,24], hydrophobic associations [25,26], dipole–dipole interactions [27], host-guest complex interactions [28], metal ions chelation [17], and polymer-nanoparticle interfacial bonds [23,29]) or several types (e.g., hydrogen bonds plus hydrophobic associations [30], hydrophobic associations plus ionic interactions [31], hydrogen bonds plus ionic interactions [32], π–π stacking plus hydrogen bonds [33], and hydrogen bonds plus ion-dipole interactions [34,35]) of non-covalent bonds to a covalent network has been demonstrated as a valid method to fabricate a tough hydrogel.

### 2.2. Highly Stretchable Polymer Network

#### 2.2.1. Polymer-Intercalated Nanocomposite Hydrogel

A homogenous, tough, and stretchable nanocomposite hydrogel has been prepared by polymer intercalating into disk-shaped nanoclay sheets [18] (Figure 1c). The initiator was adsorbed onto nanoclay surface and initiated the polymerization in situ. Multiple initiators could be adsorbed onto one nanoclay sheet. Thus, the polymers were intercalated into, and non-covalently end-coupled by, the nanoclay sheets. The polymer chains between nanoclay sheets were proportional to the distance between nanoclay sheets and with a considerably narrow distribution in length [36]. The homogenous structure of hydrogel ensures the polymer chains are evenly stretched to dissipate energy during deformation. This fabrication strategy has been extended to design tough nanocomposite hydrogels based on various nanoparticles which include polydopamine coated nanoclay [37], nanocrystalline cellulous [38], chitin nanocrystals [39], and graphene oxide nanosheets [40,41,42]. Non-covalent crosslinks, such as ionic interactions [40,41] and hydrogen bonds [37], were incorporated into the nanocomposite hydrogel to further toughen the network.

#### 2.2.2. Elastomer-Like Protein-Based Hydrogel

A tough hydrogel was constructed by crosslinking proteins containing ferredoxin-like (FL) folded domains [19]. This FL domain consists of two terminal force-bearing β-strands which are next to each other and arranged in an antiparallel form. Upon stretching, the two β-strands dissociate in an unzipping fashion and the hydrogen bonds and hydrophobic associations between the two β-strands are broken, contributing to the toughness of the hydrogel.

Another example is to crosslink elastin-like recombinant proteins [43]. This protein contains eight elastin-like domains assembled by hydrophobic associations. Each domain is comprised of five or ten pentapeptides which undergo metal coordination crosslinking. When the hydrogel is stretched, the elastin-like domains disassemble, and the metal coordination crosslinks are broken to dissipate the fracture energy (Figure 1d).

### 2.3. Double-Network Hydrogel

DN is an IPN consisting of a rigid, yet brittle, first network and a soft, but mechanically weak, second network (Figure 1e). These two networks are not suitable for mechanical loading in and of themselves. However, their unique combination yields a fracture-resistant DN hydrogel that exhibits surprisingly high mechanical properties (2–3 orders of magnitude higher than those of the two individual networks under both tensile and compressive modes) [20]. Despite having high water content (65–95 wt %), DN hydrogels exhibit fracture toughness similar to those of solvent-free rubbers and connective tissues (i.e., cartilage and ligament) [44,45]. These incredibly high mechanical properties can be replicated in various combinations of polymer pairs, ranging from synthetic acrylate polymers to biopolymers (e.g., gelatin and cellulose) [20,46,47]. It is believed that the stiff first network is responsible for the elevated strength while the ductile second network prevents macroscopic crack propagation through viscous dissipation [44,48]. Elevated toughness is observed at unique ratios of the crosslinking densities and mole ratios between these two networks [20]. Increased physical entanglement [49], molecular association [50,51], and covalent interconnection [52] between the two networks and the structural heterogeneity of the DN [20] may also play a role. Webber et al. [53] correlated the energy dissipation of DN during mechanical loading to the breakage of covalent bonds in the first network when the hydrogel deformed at a strain >20% [53,54,55]. Introducing nanoparticle [56,57] and micro-scale fibrous networks [58,59] into DN further toughens DN hydrogels. When the first network is crosslinked by non-covalent bonds (i.e., ionic interactions [60,61,62], metal ions chelation [63], hydrophobic associations [64], and hydrogen bonds [65,66]), DN hydrogels are imparted with the ability to self-heal, extensive stretchability, injectability, and printability, which broadens the application of these tough hydrogels.

## 3. Tough Hydrogels as Tissue Adhesives

Tissue adhesives play an important role in suture-less wound closure [67], tissue repair [68], immobilization of therapeutic drugs [69], cells [70,71], and medical devices [37,72,73]. Tissue adhesives can alleviate pain and discomfort associated with mechanical perforating devices (e.g., sutures, tacks, and staples) [74,75] by simplifying complex surgical procedures. In addition to exhibiting excellent biocompatibility and strong adhesion to tissue, tissue adhesives are required to resist repeated loading. In this section, recent strategies to fabricate tissue adhesives using tough hydrogels are introduced.

### 3.1. Biomimetic Adhesive Hydrogels

#### 3.1.1. Marine Mussel-Inspired Adhesive Hydrogels

**Figure 2:**
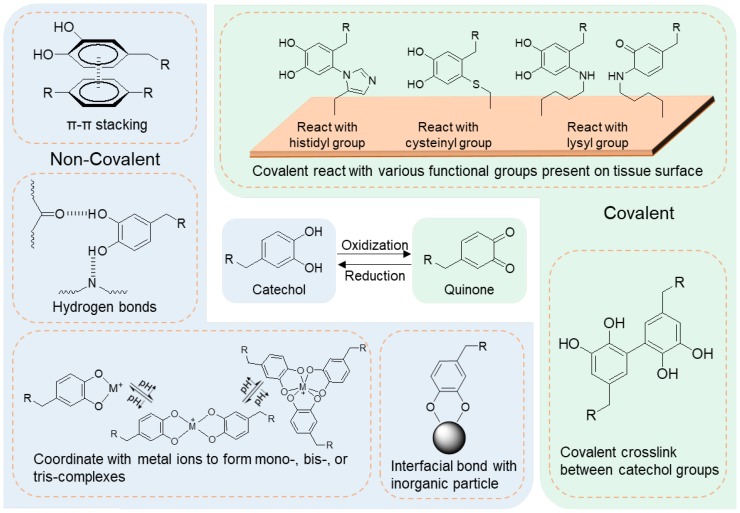
Non-covalent and covalent catechol chemistries (adapted from [76], copyright 2016 Springer).

Marine mussels secure themselves to various organic and inorganic surfaces in a wet and turbulent environment using a series of adhesive proteins. It has been verified that an amino acid, 3,4-dihydroxyphenylalanine with a catechol functional group, plays an important role in adhesion to wet surfaces. Catechol can undergo various non-covalent interactions (Figure 2), which include π–π stacking, hydrogen bonds, and coordination with metal ions. Additionally, catechol becomes highly reactive when it is oxidized, resulting in crosslinking between multiple catechol groups or between catechol and a nucleophilic group (e.g., histidyl, cysteinyl, and lysyl groups found on tissue surfaces) to form cohesive and interfacial covalent bonds.

The non-covalent chemistry of catechol has been utilized to toughen hydrogels. Incorporating catechol into non-adhesive synthetic (e.g., poly(ethylene glycol) (PEG), acrylate, and acrylamide polymer) and biological polymers (e.g., gelatin, chitosan, and alginate) has enabled these materials to bond to tissue covalently and non-covalently [77].

##### Catechol-Containing Hydrogels Covalently Bonding to Tissue

Catechol-containing tissue adhesives rely on the oxidation-mediated crosslinking of catechol for curing and tissue binding. To enhance the toughness of these hydrogels, investigators have introduced various non-covalent crosslinks to create dual-crosslinked networks. Catechol-modified gelatin formulated with ferric (Fe^3+^) ions has demonstrated 24-fold higher adhesive strength to porcine skin and cartilage surfaces when compare to that of commercial fibrin glue [78]. Fe^3+^ ions form coordination bonds with catechol and contributed to increased rate of curing and enhanced toughness and adhesive properties. Incorporation of poly(lactic-*co*-glycolic acid) [23] or silica-based [79,80] nanoparticles into catechol-containing hydrogels has been shown to increase the adhesive property by 2–3 folds. The nanoparticles provide interfacial binding sites for catechol, introducing the dissipative non-covalent interactions into the network. Introducing hydrophobic segments (e.g., polycaprolactone [81] and polypropylene oxide (PPO) [82]) to the backbone of catechol-containing hydrogels has demonstrated up to a 20-fold increase in adhesive strength. The hydrophobic segments self-assembled in the presence of water and contributed to the energy dissipation via chain slippage and disentanglements under loading. Incorporation of gelatin microgels [83] or chitin nanocrystals [29] into catechol-containing hydrogel introduces extra non-covalent crosslinks to the network, contributing to about 2–3-fold increases in adhesive strength.

The synergistic effect of catechol-metal ion coordination and other non-covalent interactions is commonly employed in tough tissue adhesive formulations. One example is that a hydrophobic segment containing micelle was incorporated into a polymer with catechol side groups, which chelated Fe^3+^ ions for the formation of coordination bonds [84]. This hydrogel exhibited excellent toughness, pH/thermo-dependent self-healing behavior, and adhesive property to multiple mice tissues (i.e., tumor, subcutaneous tissue, muscle, heart, liver, spleen, lung, and kidney). Another recent approach involved compositing catechol-containing network with collagen and hydroxyapatite nanoparticles [85]. The nanocomposite polymer network was toughened by catechol forming an interfacial bond with the ionic surface of the hydroxyapatite nanoparticles and fibrillar collagen serving as an internal physical network. This system exhibited adhesive strength that were 6–12-fold higher when compared to commercial available tissue adhesives, when bond to porcine skin in the presence of blood.

This variety of tissue adhesives exhibited good bulk mechanical property, tissue adhesive property, and biocompatibility, demonstrating very promising potential for medical applications including internal tissue adhesion, sealing, and hemostasis.

##### Polydopamine-Based Adhesives Non-Covalently Bonding to Tissue

In a basic aqueous environment, catechol containing a free amine group (i.e., dopamine) autoxidizes and polymerize to form the so-called polydopamine [86]. Polydopamine contains high concentrations of free catechol groups, which preserve its ability to form both non-covalent and covalent interactions. The incorporation of polydopamine into polyacrylamide (PAAm) covalently crosslinked network imparted the hydrogel with adhesive property to human skin [33]. Polydopamine also toughens the network through the formation of hydrogen bonds, π–π stacking, and hydrophobic associations between the catechol groups.

**Figure 3:**
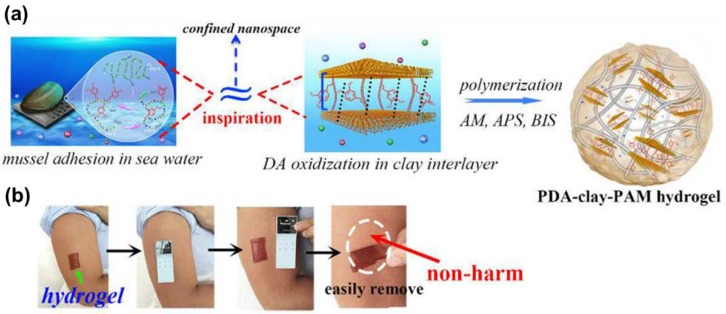
(**a**) Schematic of hydrogel formed by polymer intercalating into nanoclay sheets with polydopamine. (**b**) Photographs of hydrogel that sticks to human skin and can be easily removed (reproduced from [37], copyright 2017 ACS).

Inspired by the polymer-intercalated nanocomposite hydrogel fabrication strategy, a tough polydopamine-clay-PAAm adhesive hydrogel was prepared [37]. Polydopamine was formed between the layers of disk-shaped nanoclay. Then, acrylamide (AAm) monomers were polymerized in situ on the surface of clay sheets (Figure 3a). This polymer-intercalated nanocomposite hydrogel showed high toughness and extensibility, and repeatedly adhered to human skin with minimal skin damage (Figure 3b). It also demonstrated excellent performance as a wound dressing when incorporated with epidermal growth factor in rat full-thickness skin defect experiments. A type of conductive adhesive hydrogel was prepared by a similar procedure using carbon nanotubes (CNTs) [72] or graphene oxide [73]. These hydrogels may be used as wearable or implantable bioelectronic devices. Additionally, polydopamine itself can be prepared in the form of nanoparticles which served as crosslinking points to polymer chains in a hydrogel [56]. The near-infrared responsiveness of polydopamine nanoparticle provides this hydrogel with potential for chemical and physical therapy applications, while synergistically working with a thermo-sensitive polymer matrix.

#### 3.1.2. Tannic Acid-Based Adhesive Hydrogels

**Figure 4:**
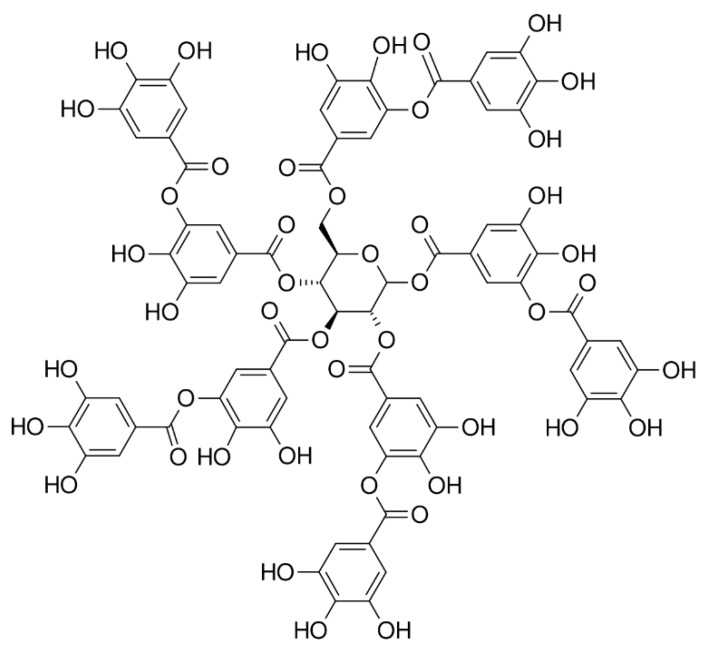
Chemical structure of tannic acid.

Plant-derived polyphenols, such as tannic acid, can serve as biocompatible and low-cost alternative adhesive moieties for designing robust adhesive hydrogels [87]. Tannic acid is a molecule containing five catechol and five pyrogallol (tri-hydroxyphenyl) groups (Figure 4). Similar to catechol, pyrogallol forms both non-covalent interactions and covalent crosslinks. The large amount of phenol groups present on tannic acid enables dense crosslinking and strong tissue adhesion of tannic acid-containing hydrogel. Additionally, tannic acid-containing adhesive exhibited an antimicrobial property, which is likely due to the ability of tannic acid to remove metal ions from bacterial cell walls and membranes [88]. Tannic acid forms strong non-covalent interactions with proteins (e.g., thrombin [89] and elastin [90]) and DNA [91], which has been utilized to fabricate tough and extensible adhesive hydrogels with superior adhesive and hemostatic properties. 

#### 3.1.3. Oyster-Inspired Adhesive Hydrogels

Oysters bind themselves together into clusters to construct a very strong protective reef-like structure via a biomineralized adhesive which contains about 50 wt % of calcium carbonate (CaCO_3_) and 11 wt % of crosslinked acidic proteins [92,93]. Inspired by the mineralized oyster adhesive, an injectable adhesive hydrogel was constructed by using CaCO_3_ nanoparticles to crosslink poly(acrylic acid) (PAA) [94]. The generated hydrogel showed comparable adhesive strength to mussel-inspired adhesives in both dry and wet conditions.

### 3.2. Nanocomposite Hydrogels as Tissue Adhesives

The synthetic disk-shaped nanoclay plays an important role in constructing tough and stretchable hydrogels via non-ionic polymers intercalating into the interlayers of nanoclay sheets (see Section 2.2.1). Addition of a second type of nanosheet, functionalized-boron nitride, into polymer-intercalated nanoclay nanocomposite hydrogel introduces additional non-covalent interactions which include hydrogen bonds, metal ion chelation, π–π stacking, and cation–π interactions [95]. Due to the high concentration of non-covalent interactions, this hydrogel exhibited a self-healing capability and strong adhesion to human skin, as well as various solid surfaces (e.g., metal, glass, wall, plastics, rubber, and ceramic).

Additionally, hydrogels can be constructed by intercalating a dendrimer with cationic end groups [96] or polymer with cationic substituents [97] into the interlayers of nanoclay sheets. Nanoclay sheets display high concentration of negative charges on the surface, which are able to form strong electrostatic interactions with cationic groups. These hydrogels are non-covalently crosslinked and different hydrogel pieces could be rapidly glued together through the supramolecular electrostatic interactions.

**Figure 5:**
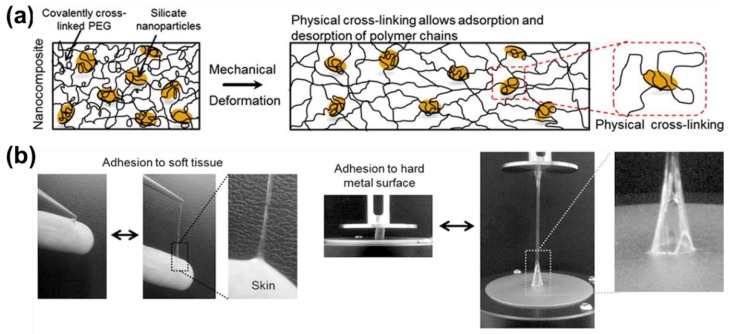
(**a**) Schematic of poly(ethylene glycol) (PEG) hydrogel incorporated with nanoclay. PEG chains form non-covalent bonds to nanoclay. (**b**) Photographs of hydrogel that is stretchy and sticks to human skin and metal surfaces in an elongated state (reproduced from [34], copyright 2011 Elsevier).

Another strategy to fabricate a tough hydrogel based on the disk-shaped nanoclay is to create promoted interfacial interactions (i.e., hydrogen bonds and ion-dipole bonds) between the nanoclay and the covalently crosslinked polymer (e.g., poly(ethylene oxide) (PEO)_99_-PPO_65_-PEO_99_ (Pluronic F127) and PEG with a molecular weight from 12 to 35 kDa) [34,35] (Figure 5a). The linear polymer was end-modified to prepare mono-acrylate macromer or di-acrylate macro-crosslinker, which was able to be covalently crosslinked to form a hydrogel. The addition of nanoclay to the covalent network effectively increased its toughness, stretchability, and mammalian cell adhesion. It also demonstrated almost two-fold higher adhesive strength to human skin and hard surfaces, such as glass and metal (Figure 5b), compared to the nanoclay-free hydrogel. These nanocomposite hydrogels have the potential to be developed as tissue matrices for soft substrates as wound dressings and sealants, or hard substrates as dental and orthopedic repair materials.

### 3.3. Tough and Stretchable IPN as a Tissue Adhesive

IPN, composed of calcium (Ca^2+^) ion crosslinked alginate and a loosely-crosslinked PAAm covalent network, exhibited high toughness and stretchability [98]. Crosslinking the alginate/PAAm IPN over a pre-functionalized surface can covalently bond the hydrogel to the substrates. This hydrogel demonstrated strong adhesion to elastomer [98], as well as diverse non-porous solid surfaces, including glass, silicon, ceramics, titanium, and aluminum [99,100]. This strategy may be used for embedding electronics and sealing stretchable electronics in hydrogels.

**Figure 6:**
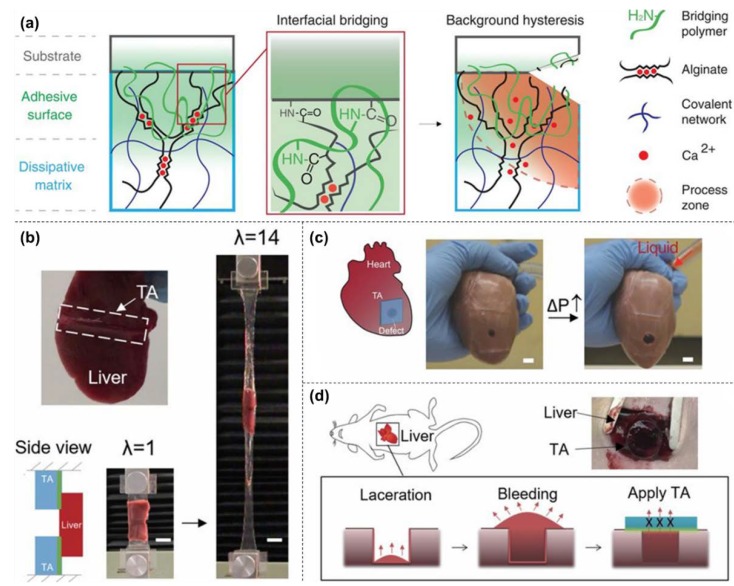
(**a**) Schematic of interpenetrating network (IPN) hydrogel coated with tissue adhesive polymer. (**b**) Photographs of hydrogel gluing liver tissue in an elongated state. (**c**) Photographs of hydrogel functioning as a tissue sealant and (**d**) hemostatic wound dressing (reproduced from [101], copyright 2017 Science).

By coating a polymer with a high concentration of primary amine onto the alginate/PAAm IPN, the hydrogel demonstrated strong adhesion to wetted tissue surfaces (Figure 6a) [101]. This polymer can adhere to tissue via physical penetration of polymer chains, primary amine-mediated electrostatic interactions, and covalent reaction with carboxylic acid groups present on the tissue surface. The modified IPN exhibited integrated properties of strong adhesion and large deformability. It could serve as a tissue adhesive, heart sealant, and hemostatic dressing even in the expanded state (Figure 6b–d) and showed a two orders of magnitude improvement in adhesion energy than commercial sealants.

## 4. Tough Hydrogels for Tissue Engineering

Tissue engineering combines cells, scaffold, and growth factors to construct functional artificial tissues or organs for therapeutic or research purposes. A tissue engineering scaffold works as a template for tissue regeneration by providing cells with chemical cues and mechanical support. Hydrogels have been widely used as tissue engineering scaffolds [102,103,104]. However, hydrogels are mechanically weak and sometimes cannot provide the desired stiffness for cell differentiation and function [105,106,107,108]. This section discusses the design and utilization of tough hydrogels as acellular and cell-laden tissue engineering scaffolds. We highlight the biocompatibility concerns in these applications as well as recent advances in using 3D-printing technology to construct tough hydrogels. Examples for using tough hydrogels in cartilage, cornea, and cardiovascular tissue engineering are reviewed.

### 4.1. Tough Hydrogel Implementation Methods

#### 4.1.1. Tough Hydrogel as an Acellular Scaffold

A tough hydrogel can potentially function as an acellular scaffold for tissue repair [109,110,111,112]. One of the key criteria for designing tissue engineering scaffolds is to minimize cytotoxicity associated with the unreacted monomers. For example, synthetic polymer, such as PAAm, is popular for tissue engineering [59,113,114]. However, the AAm monomer is a known neurotoxin [115] and a risk factor for several cancers [116,117]. Darnell et al. [113] have systematically quantitated the change of AAm concentration over time in an alginate/PAAm IPN hydrogel designed for tissue engineering. A mild accumulative cytotoxic effect was observed after long-term static cell culture, potentially due to the presence of unreacted AAm. Additionally, synthetic polymer, such as poly(*N*,*N*-dimethyl acrylamide) (PDMAAm) and PEG, are bioinert and unable to promote cell adhesion and tissue regeneration [118,119]. Thus, it is necessary to incorporate adhesive components that cells can recognize (e.g., to integrate ECM proteins or integrin recognition peptides) into a synthetic polymer network.

**Figure 7:**
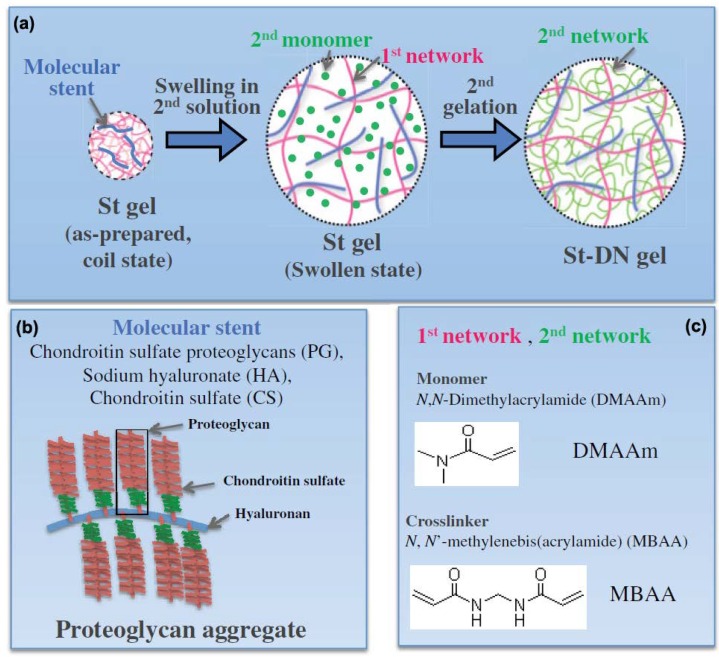
(**a**) Schematic of the formation of a double network by incorporating proteoglycan aggregate stent (St) molecules into the neutral networks. (**b**) Schematic of the St molecule, proteoglycan aggregate. (**c**) Schematic of the monomer and crosslinker used to construct the neutral networks (reproduced from [119], copyright 2014 Wiley).

Zhao et al. [119] used a molecular stent technique to create a DN hydrogel composed of charged biomacromolecules (i.e., proteoglycan aggregate, hyaluronic acid (HA), and chondroitin sulfate) and neutral PDMAAm networks (Figure 7). The highly-charged proteoglycan greatly extended the first PDMAAm network due to charge repulsion to promote the diffusion of the second network precursor solution, which ensures the formation of a tough DN [20]. The incorporation of proteoglycan also promoted human coronary artery endothelial cell adhesion and proliferation. This molecular stent method can be used to combine various biomacromolecules found in cartilage tissue with synthetic polymers to generate tough hydrogels suitable for cartilage repair.

Incorporating a synthetic polymer that controls charge distribution along the polymer chain can also enhance cell adhesion. Chen et al. [120] developed a triple network hydrogel, where a copolymer consisted of poly(2-acrylamido-2-methyl-propane sulfonic acid sodium salt) (PNaAMPS) and PDMAAm blocks were incorporated into a mechanically-robust PNaAMPS/PDMAAm DN hydrogel as the third network. The negatively-charged PNaAMPS promoted fibronectin absorption [121], which subsequently facilitated cell adhesion and proliferation. By adjusting the composition of the di-block copolymer in the third network, bovine fetal aorta endothelial cell adhesion and spreading rate were controlled. Elevated concentrations of negatively-charged polymer reduced the mechanical property of the hydrogel due to excessive swelling. The neutral PDMAAm polymer was, therefore, incorporated to mitigate this effect.

#### 4.1.2. Tough Hydrogel as a Cell-Laden Scaffold

Using an acellular scaffold to repair injured tissue is a straightforward and time-efficient strategy. However, it requires that the patient’s progenitor cells are able to migrate, proliferate, and differentiate in the scaffold for tissue regeneration. Individuals with severe trauma or diseases, such as diabetes or angiogenesis defects, may not be suitable for this therapy. With the development of stem cell technologies, cell-laden scaffolds can potentially solve this issue while eliciting minimal immune responses [122,123]. However, a complicated cell encapsulation procedure may reduce cell viability [124]. Therefore, using facile cell-embedding procedures and introducing cues (e.g., ECM components or small peptides derived from ECM) that can enhance cell viability are necessary.

Shin et al. utilized gelatin to develop two tough hydrogels (a DN hydrogel [124] and a microgel-reinforced (MR) hydrogel [125]) that were encapsulated with cells. The DN used gellan gum methacrylate (GGMA), and gelatin methacrylate (GelMA) to construct the first and second network, respectively. Cells were pre-mixed within the GGMA precursor solution to prepare the cell-laden first network via ultraviolet (UV)-initiated crosslinking. The first network was soaked in GelMA precursor solution to prepare the cell-laden DN via a second UV-initiated crosslinking procedure. To create the MR, a microgel composed of GGMA was incorporated into the GelMA precursor solution pre-loaded with cells. Then the cell-laden MR hydrogel was prepared by one-step UV-initiated crosslinking. Although the chemistry and mechanical property of these two hydrogels are very similar, the MR scaffold facilitated osteogenic differentiation of the encapsulated MC3T3-E1 preosteoblasts more effectively than DN scaffold. This likely due to reduced UV exposure in MR [126]. Additionally, the microgels may act as additional crosslinking points which limit the swelling of MR to provide cells with better mechanical cues during cell culture. Moreover, cells had direct access to the gelatin component in MR, which enhanced cell adhesion, metabolic rate, and osteogenic differentiation.

**Figure 8:**
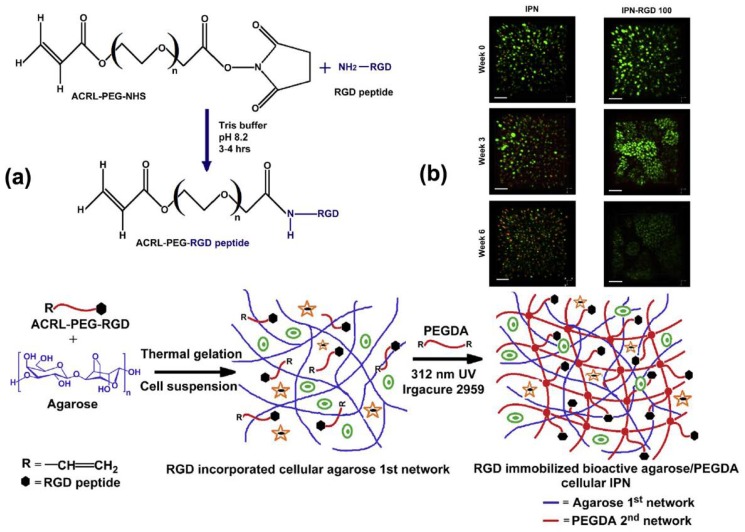
(**a**) Schematic of the formation of a cell-laden interpenetrating network (IPN) hydrogel immobilized with RGD peptide sequences. (**b**) Illustration of chondrocyte outgrowth in a hydrogel with and without RGD via live/dead staining (reproduced from [128], copyright 2014 Elsevier).

Incorporation of small peptides derived from ECM (e.g., Arg-Gly-Asp (RGD)) into the hydrogel can promote cell adhesion, proliferation, and ECM deposition [127]. Ingavle et al. [128] incorporated chondrocytes into a tough agarose/PEG IPN immobilized with RGD for cartilage tissue repair (Figure 8a). The chondrocytes encapsulated in IPN with RGD exhibited a higher viability, proliferation rate, and ECM (i.e., glycosaminoglycan and collagen) deposition rate than those in IPN without RGD. Collectively, the chondrocytes in the IPN with RGD demonstrated higher cell outgrowth (Figure 8b), resulting in better integration of the regenerated tissue with the surrounding tissues. Unlike biomacromolecules, small peptides can interact with cells more specifically (e.g., RGD’s effects are through integrin to promote cell adhesion [129] or matrix metalloproteinase to facilitate tissue remodeling [130]). Thus, this concept may be useful to design tough hydrogels with specific bioactivities and controllable biodegradation property.

To achieve both fast curing and incorporation of bioactive moieties into a tough hydrogel, Truong et al. [131] developed a chitosan/PEG DN hydrogel using one-pot synthesis approach. The first network (formed through the thiol-alkyne addition) and the second network (formed through tetrazine-norbornene addition) were formed simultaneously in one mixture. The crosslinking process occurred in a mild aqueous condition in phosphate-buffered saline (PBS) and cell culture medium and the crosslinking reaction was completed within 3 min. Mesenchymal stem cells (MSCs) were pre-loaded into the precursor solutions and the solidified DN hydrogel demonstrated excellent cell viability and homogeneous cell distribution. This work demonstrates a universal strategy to prepare tough DN hydrogels with tunable mechanical property and ability to chemically link biomolecules using click chemistry.

#### 4.1.3. 3D-Printed Tough Hydrogel

Advanced manufacturing techniques have been adopted to construct tough hydrogels for tissue engineering, which include electrospinning [132,133], multilayer fabrication [134,135], 3D-weaving [59,136,137], and 3D-printing technologies [138,139]. Among these techniques, 3D-printing is particularly useful for personalized medicine applications to generate items with customized complex micro- and macro- structures, or with direction-dependent properties. Such structures can greatly minimize the transplantation mismatch and better mimic the native tissue architecture. This section reviews 3D-printed tough hydrogels for biomedical applications.

##### Design of Ink for Tough Hydrogel Printing

There are two key requirements for using hydrogel precursor solutions as 3D-printing ink. The first requirement is that the viscosity of the precursor solution need to be suitable for it to flow within the printer and to be extruded through the printer nozzle. Secondly, the precursor solution needs to rapidly cure during the printing process.

To enable a tough alginate/PAAm IPN hydrogel for 3D-printing, Bakarich et al. [140] optimized the viscosity of the precursor solution for printing by adjusting the alginate concentration. The solution cures within 90 s of UV irradiation. To print a kappa-carrageenan/poly(ethylene glycol) di-glycidyl ether (k-CG/PEGDGE) IPN hydrogel, changing temperature was used to achieve hydrogel fast curing [141]. Controlling the degree of covalent crosslinking between the epoxy group found on PEGDGE and the amine functional group found on kCG was used to adjust the viscosity of the ink. The yydrogel precursor solution was printed through a heated printer nozzle with a temperature of 50.4 °C, which is the thermal gelation temperature of k-CG, onto a cooled substrate at 21 °C. At the same time, the epoxy-amine crosslinking reaction continued in situ to complete the curing process.

**Figure 9:**
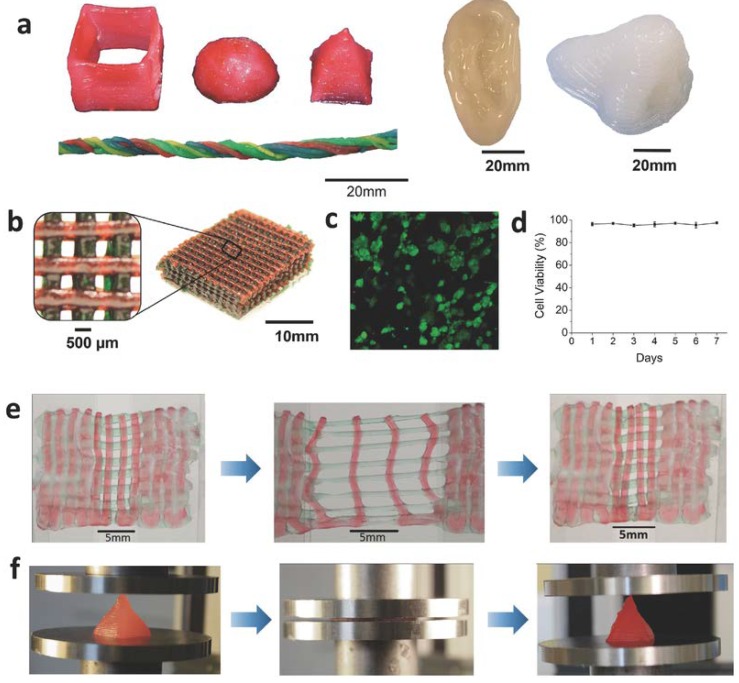
(**a**) Photographs of 3D-printed hydrogels with shapes of a hollow cube, hemisphere, pyramid, twisted bundle, artificial ear, and nose. (**b**) A photograph of scaffold frame printed by the hydrogel in a meshed fashion. (**c**,**d**) Live/dead assay and cell viability of human embryonic kidney (HEK) cells in the scaffold. (**e**) Photographs of stretching and recovery of the printed hydrogel mesh. (**f**) Photographs of compression and recovery of the printed hydrogel pyramid (reproduced from [142], copyright 2015 Wiley).

Hong et al. [142] 3D-printed an alginate/PEG IPN with a nanosilicate, LAPONITE^®^ (Figure 9a). LAPONITE^®^ tuned the viscosity of the ink and made it highly shear-thinning and suitable for printing. Once the ink was extruded, the shear rate decreased so rapidly that the ink became too viscous to deform. The hydrogel was printed in a meshed structure to create a cell-laden scaffold (Figure 9b). Human embryonic kidney (HEK) cells were introduced into the scaffold and the cell exhibited >95% viability even after seven days in culture (Figure 9c,d). The printed hydrogel mesh exhibited high toughness under tensile and compressive loads (Figure 9e,f). Another example of using LAPONITE^®^ to tune the ink viscosity was reported in Yang et al.’s work [143], where DN hydrogel-based artificial menisci were printed. LAPONITE^®^ optimized the viscosity of the precursor solution of first network. The second network was introduced to the printed meniscus-shaped first network afterwards.

It is important to note that a 3D-printed hydrogel may be mechanically weaker when compared to a hydrogel with the same composition prepared through the traditional solvent casting approach. This is likely due to the generation of defects during the printing process. Using an ink with higher viscosity can potential limit the hydrogel deformation during printing so as to improve the printing resolution, which may resolve this problem [140].

##### 3D-Printed Hybrid with Tough Hydrogel Infused

Given that it may be time-consuming to develop a new 3D-printable tough hydrogel, an alternative involves developing a composite that combines a 3D-printed thermoplastic-fiber mesh and a tough hydrogel network. This approach combines a well-established printing technology with hydrogels of known toughness without the need to adjust the hydrogel formulation and curing process for printing. A 3D-printed polylactic acid (PLA) was embedded within a cell-laden alginate/PAAm IPN hydrogel matrix [58]. By varying the density of the PLA fibers, the mechanical property of the composite was optimized. The pattern and density of the fibers can be adjusted to facilitate tissue regeneration by providing the desired anisotropic topography for cell growth.

### 4.2. Examples of Tough Hydrogels for Tissue Engineering

#### 4.2.1. Cartilage Tissue Engineering

Cartilage is an avascular tissue which is difficult to heal after injury. Surgical repair of cartilage is particularly important to facilitate its healing and prevent further tissue necrosis and pain. A highly wear-resistant DN hydrogel was fabricated for cartilage repair as an acellular scaffold [144]. In a rabbit subcutaneous implantation study, the hydrogel maintained excellent mechanical properties and exhibited minimal change in water content (less than 3%) after six-week implantation [145]. Only a mild inflammatory response was observed at week 1 and no significant inflammation was observed after that [146]. The friction coefficient between DN and human cartilage was significantly lower than that of cartilage-to-cartilage contact [147]. In the rabbit osteochondral defect model study, the defect treated with DN demonstrated better regeneration of new cartilage tissue when compared with groups without treatment [148]. However, the regenerated tissue is very similar, but not identical, to the natural hyaline cartilage based on microarray gene profiling [149].

One approach to improve acellular scaffolds for cartilage tissue engineering involves loading the scaffolds with chondrocytes or MSCs that can be induced to differentiate into chondrocytes. Excellent chondrocyte viability and chondrogenic differentiation of the encapsulated MSCs has been confirmed in both fibrin/HA [122] and polyglycolic acid/HA [123] IPN hydrogels. Many published studies have explored the potential of using cell-laden tough hydrogels for cartilage repair [128,150,151,152,153,154,155,156]. However, incorporation of cells may lead to a reduction in mechanical strength of tough hydrogel [157], which should be a concern in the practical applications.

#### 4.2.2. Cornea Tissue Engineering

A non-degradable tough hydrogel with good optical properties and high diffusion coefficients for nutrient molecules is potentially a good candidate for artificial cornea. There are two Food and Drug Administration-approved artificial cornea currently on the market: Boston KPro (poly(methyl-methacrylate) (PMMA)-based material) and AlphaCor (poly(2-hydroxyethyl methacrylate) (PHEMA)-based hydrogel). However, they are only allowed to be used on patients with repeated transplant rejections [158,159]. A synthetic cornea that can integrate well with the surrounding tissues and allows corneal epithelialization is still needed.

**Figure 10:**
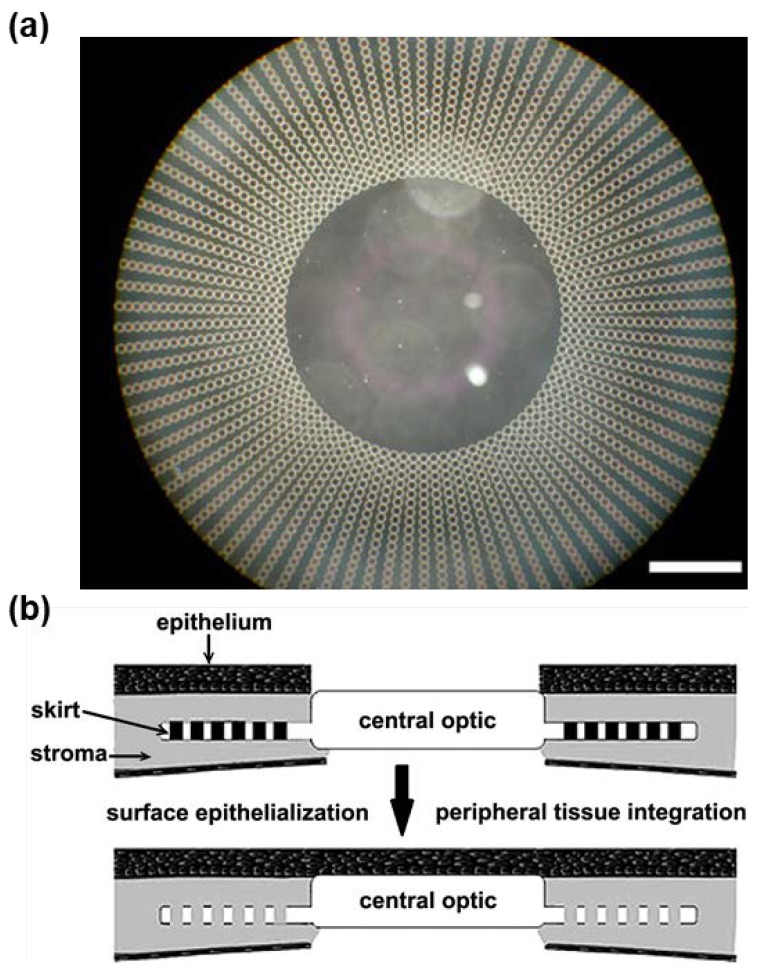
(**a**) A photograph of the artificial cornea. Scale bar: 1300 μm. (**b**) Ideal schematic representation of the implanted artificial corneal (reproduced from [161], copyright 2007 Springer).

Tan et al. [160] designed an artificial cornea with a mechanically strong PEG/PAA IPN hydrogel as the core and a microperforated poly(hydroxyethyl acrylate) (PHEA) hydrogel as the periphery (Figure 10a), which was used to anchor the implant. Both core and periphery were demonstrated to withstand the upper limit of the optical pressure. The core exhibited similar level of light transmissibility, refractive index, and glucose diffusion coefficient as those of a human cornea. When collagen was chemically bound to the surface of the artificial cornea, primary rabbit corneal epithelial cells were successfully cultured on the core surface, which reached confluence after 48 h (Figure 10b). Additionally, primary rabbit corneal fibroblast cells successfully adhered and proliferated to confluence on the periphery surface within 24 h (Figure 10b). These results indicate that the integration of this artificial cornea with stroma through fibroblast ingrowth might be possible. This artificial cornea was intrastromally implanted in rabbit, where the implant remained clear and the epithelium structure was well-maintained without other adverse effects after 14 days [161].

Oelker and Grinstaff [162] constructed a bi-layered artificial cornea consisting of a mechanically tough poly(2-hydroxyethyl methacrylate-*co*-methacrylic acid) (P(HEMA-*co*-MAA)) hydrogel layer and a dendritic PEG-based hydrogel layer. Both layers exhibited over 88% light transmissibility. The surface of the P(HEMA-*co*-MAA) layer was modified with collagen to demonstrate the support of re-epithelialization, while the surface of the PEG layer was modified with RGD to demonstrate the support of fibroblast adhesion and proliferation to confluence during a four-week culture period.

These results demonstrate good examples of using latest fabrication techniques (e.g., photolithographic patterning) and surface modifications to tune the material properties of tough hydrogels, which is promising to develop a new artificial cornea.

#### 4.2.3. Cardiovascular System Tissue Engineering

Collagen hydrogel is the most widely used material for constructing a scaffold for blood vessel and heart tissue replacement. However, conventional collagen hydrogels are mechanically weak. Once cells adhere and proliferate within the collagen scaffold, the structure tends to undergo severe compactions. To resolve this problem, Munoz-Pinto et al. [163] developed a cell-laden collagen/PEG IPN hydrogel with encapsulated MSCs. This method significantly improved the mechanical property while limited cell-induced degradation of the PEG network by pre-immobilizing cells into the collagen network. The encapsulated MSCs differentiated into smooth muscle cells after 14 days in culture without significant scaffold compaction. Additionally, this IPN reduced blood clotting by 40% when compared with a collagen hydrogel.

To construct micrometer-scale tissue engineered vessels, a gelatin/HA IPN was used to support the perfusion culture of human umbilical vein endothelial cells (HUVECs) [164]. HUVECs formed cylinder-shaped blood vessels (200–700 μm diameter) within the hydrogel. These vessels were demonstrated to withstand media perfusion into the artificial vessels (pressure corresponding to mean velocity of 5 mm/s and shear stress of 1 dyne/cm^2^). Remarkably, HUVEC cells in the artificial tissue generated new vessel sprouts after 3–5 days of culture. This work provides inspiration for using tough hydrogel to fabricate highly-organized pre-vascularized artificial tissues.

A cardiac tissue engineering scaffold needs to mimic the protein-based aligned nanofibrous architecture found in native cardiac tissues. The scaffold also needs to be electrically conductive and mechanically strong for contractile beating. Shin et al. [165,166] constructed a conductive nanofibrous hydrogel using CNT-embedded gelatin. The embedded CNTs reinforced the hydrogel network and improved the spontaneous synchronous beating rate and excitation threshold needed for cardiac tissue. Additionally, CNTs acted as scavengers of free oxygen radical and protected cardiac cells from damages caused by oxidative stress [166]. An electrospinning technique was used to generate a highly-aligned CNT-embedded poly(glycerol sebacate)/gelatin nanofibrous structure, which promoted the alignment of cardiomyocytes seeded on the scaffolds in the direction of fibers [133]. This work demonstrates a good example of constructing complex artificial tissues for organ-on-chip applications using a combination of tough hydrogel chemistry and modern fabrication techniques.

## 5. Tough Hydrogels for Actuators and Soft Robots

Hydrogel-based actuators and soft robots have been an active research area due to several advantages of using a soft and compliant materials for biomedical applications over traditional metal-based material [167]. Hydrogel actuators have a greater degree-of-freedom in their movements due to excellent flexibility, which is important in handling fragile tissues or biomaterials with a complex geometry. Hydrogels, in general, also have good biocompatibility due to elevated water contents. However, hydrogels need sufficient toughness so that they can repeatedly undergo large deformation during cyclic actuation and movement. In this section, we review both homogenous and bi-layered hydrogel actuators that respond to changes in electric fields, light, ionic strength, temperature, and hydraulic pressure. These hydrogels have great potential in biomedical applications, such as medical device flow regulators, electronic skin, and artificial muscle.

### 5.1. Actuation of Homogenous Tough Hydrogels

**Figure 11:**
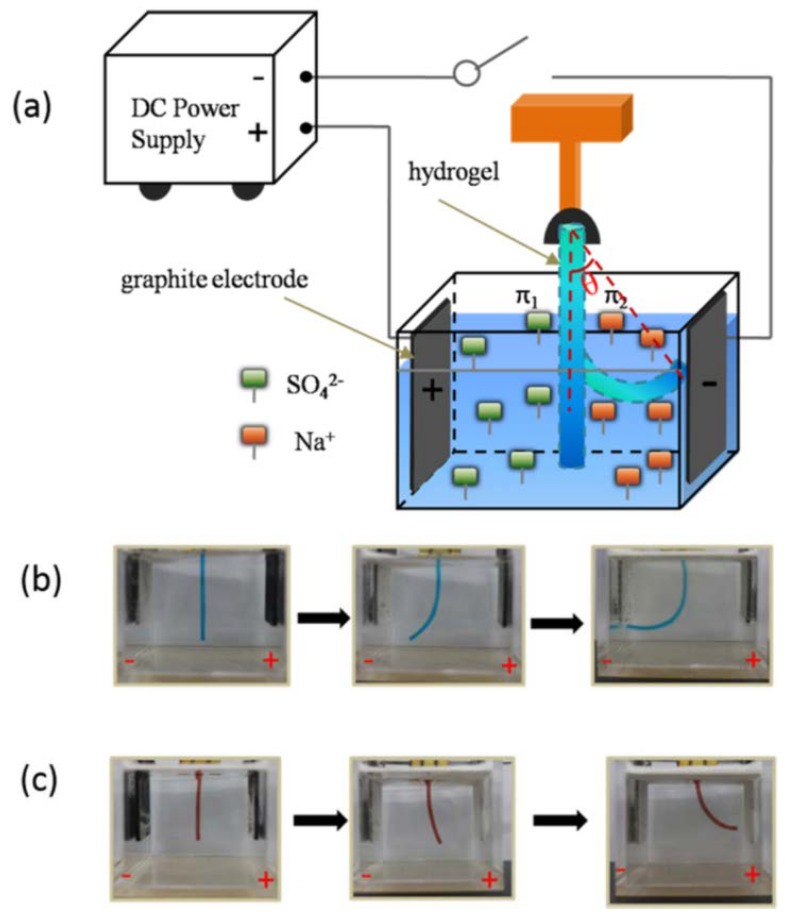
(**a**) Schematic of polyionic hydrogel submerged in between two parallel electrode plates in an electrolyte solution. (**b**) Photographs of the polyanionic hydrogel bending towards the cathode. (**c**) Photographs of the polycationic hydrogel bending towards the anode (reproduced from [171], copyright 2016 ACS).

Exposing an ionic homogeneous hydrogel to an electric field creates a gradient of ionic strength within the hydrogel [167], which leads to the hydrogel bending due to the different local osmotic pressure and swelling ratio [168]. Sun et al. [169,170] reported a polycationic hydrogel constructed with a cationic monomer, 2-(*N*,*N*-dimethylamino)ethyl acrylate quaternary ammonium salt (DMAEA-Q), and Pluronic F127 di-acrylate with elevated toughness and stretchability. Pluronic F127 di-acrylate formed nanomicelles, which served as the crosslinker to create the tough hydrogel. Similarly, a polyanionic hydrogel was prepared by copolymerizing an ionic monomer, 2-acrylamido-2-methyl-propane sulfonic acid (AMPS), and Pluronic F127 di-acrylate [171]. Li et al. [171] demonstrated the electro-responsive actuation of these polyionic hydrogels (Figure 11a). When an electric field was applied, the anions in the hydrogel moved toward the anode, causing a decrease of local ionic concentration on the cathode side. This caused the polyanionic hydrogel to bend toward the cathode (Figure 11b). Conversely, a polycationic hydrogel bent toward the anode (Figure 11c). These polyionic hydrogels exhibited reversible bending behaviors under the periodical electric fields. Morales et al. [172] used this principal to design an electro-responsive two-leg walker with polycationc and polyanionic hydrogel as each leg, respectively. The walker performed unidirectional motion on a flat surface by alternating the bending of each leg in response to the changes in the direction of the applied electric field.

**Figure 12:**
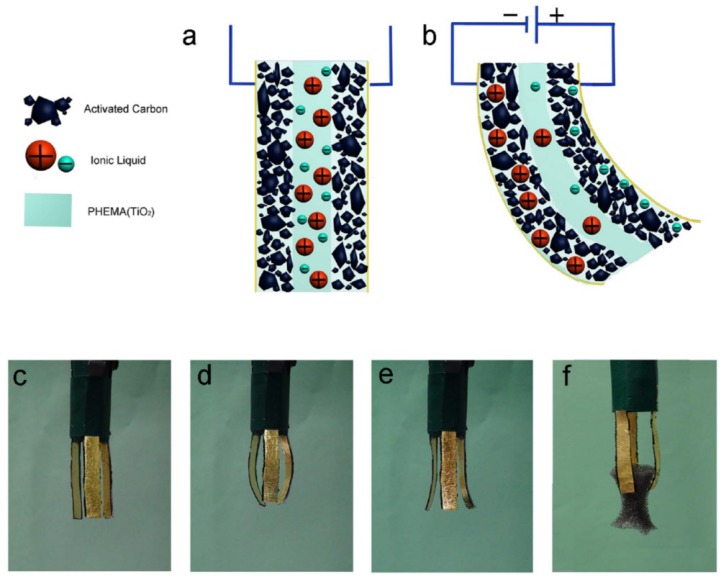
(**a**) Schematic of hydrogel containing ionic liquid sandwiched between two porous activated carbon layers. (**b**) Schematic of the hydrogel “sandwich” actuated by an applied voltage. (**c**–**f**) Photographs of a functioning manipulator constructed with multiple pieces of the hydrogel “sandwich” that can grip an object (reproduced from [174], copyright 2014 Springer Nature).

Santaniello et al. [173] reported a high-performance electro-responsive hydrogel containing polyelectrolyte segments in the matrix and embedded cellulose nanocrystals (CNCs). The incorporation of the CNCs significantly increased the mechanical property and sensitivity of the hydrogel responding to an electric field. The hydrogel was shaped like a valve and demonstrated electro-responsive flow control via the bending actuation. Liu et al. [174] introduced a nanocomposite hydrogel with confined ionic molecules for performing electro-responsive actuation. The hydrogel was prepared by in situ crosslinking of a non-ionic monomer, HEMA, in an ionic liquid, 1-butyl-3-methylimidazolium tetrafluoroborate (BMIMBF_4_), containing titanium oxide (TiO_2_) nanoparticles. The hydrogel strip was sandwiched between two porous activated carbon layers as electrodes. When a voltage was applied, the BMIM^+^ and BF_4_^−^ ions moved to the cathode and anode layers, respectively. Due to the larger size of BMIM^+^ when compared to that of BF_4_^−^, the cathode layer swelled while the anode layer shrank, causing the hydrogel to bend (Figure 12a,b). Increasing the amount of TiO_2_ nanoparticles enhanced the toughness of hydrogel under compression. A manipulator was assembled by several composite pieces of the hydrogel strip sandwiched between two gold foil layers (Figure 12c–e). This manipulator was demonstrated to grip a 0.18 g object in response to an electric field (Figure 12f) and maintain its function over a large temperature range (−10 to 100 °C).

Dalaney et al. [175] reported a photo-responsive hydrogel actuator used for controlling the fluid flow in a microfluid device. A hydrogel, constructed with a thermo-responsive polymer, poly(*N*-isopropylacrylamide) (PNIPAAm), functioned as the valve in the device. This thermo-responsive hydrogel was modified with a photo-responsive molecule, spiropyran. Initially the hydrogel swelled to block the channel. When the LED light was on, spiropyran changed its structure due to photoisomerization and reduced the lower critical solution temperature (LCST) of the hydrogel. When the LCST was below the experimental temperature, the hydrogel became hydrophobic and shrank [176], allowing the fluids to pass through the channel. 

### 5.2. Actuation of Bi-Layered Tough Hydrogels

**Figure 13:**
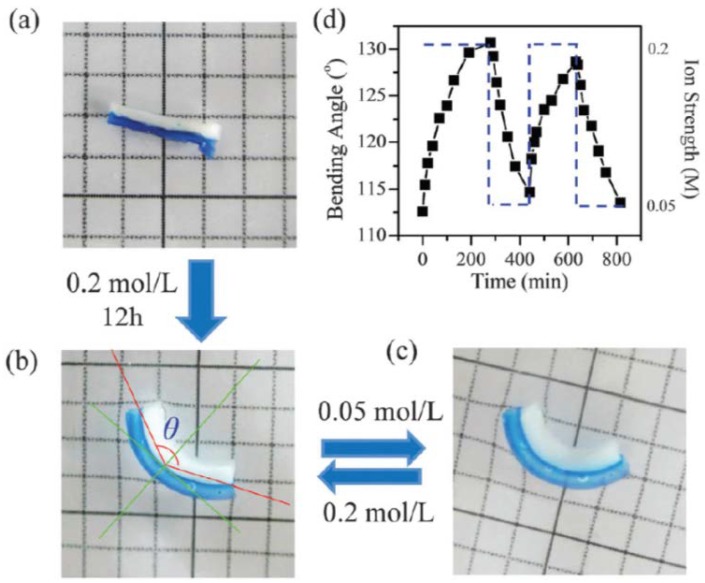
(**a**) Photograph of a bi-layered hydrogel consisting of polyanionic (blue) and polycationic (white) hydrogels. (**b**,**c**) Photographs of a bi-layered hydrogel bending towards the polycationic side in 0.2 M (**b**) and 0.05 M (**c**) NaCl solution. The bending angle (θ) is defined as shown. (**d**) The reversible actuation of hydrogel shuttling between 0.2 and 0.05 M NaCl solutions (reproduced from [178], copyright 2016 RSC).

A bi-layered actuator made of two types of metal was first described in 1925 [177]. Due to the difference in the thermal expansion coefficients between two metals, the bi-layered metal bends sharply when the temperature is increased. Similarly, researchers combined two types of hydrogels with different swelling ratios under the same condition into a bi-layered hydrogel. Liu et al. [178] designed a tough bi-layered actuator consisting of a cationic polymer-intercalated and an anionic polymer-intercalated nanocomposite hydrogels. The polyanionic layer showed a higher swelling ratio than the polycationic layer when they were submerged in the solution with a relatively low ionic strength, causing the bi-layered hydrogel to bend towards the polycationic side (Figure 13a,b). The bending angle could be further modulated by the ionic strength (Figure 13c). The reversible actuation was demonstrated by shuttling the actuator between 0.05 and 0.2 M NaCl solutions (Figure 13d).

Zheng et al. [179] fabricated a tough, thermo-responsive bi-layered hydrogel composed of aluminum (Al^3+^) ion-crosslinked alginate/PNIPAAm IPN (Al-alginate layer) and non-crosslinked alginate/PNIPAAm IPN with sodium (Na^+^) as the counter ions (Na-alginate layer). The LCST of Al-alginate layer can be regulated by the concentration of Al^3+^ ions and is significantly lower than that of Na-alginate layer. When the temperature was between the LCST of Al-alginate and Na-alginate layers, the Al-alginate layer shrank while the Na-alginate layer remained unchanged, causing the actuator to bend toward the Al-alginate layer. The bi-layered actuator exhibited reversible actuations with a maximum bending angle of 140° over several cycles. The actuator was made into the shape of a gripper, which was able to pick up and hold a 0.52 g object.

**Figure 14:**
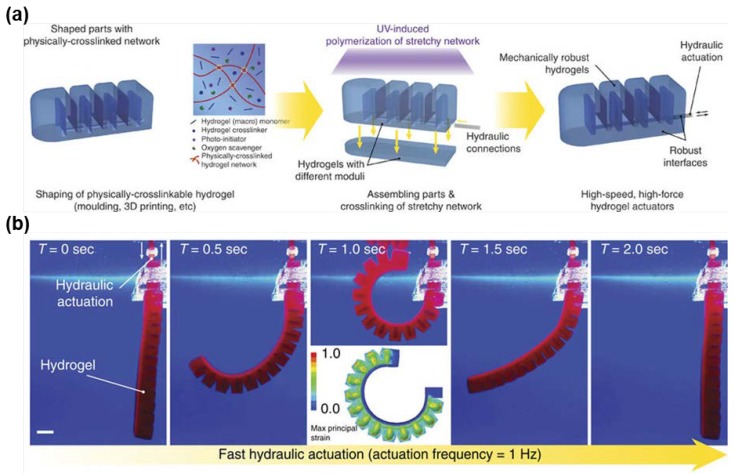
(**a**) Schematic of assembling a bi-layered hydraulic actuator consisting of a softer top layer shaped to several serial units and a stiffer bottom layer. (**b**) Photographs of the bi-layered hydrogel actuated by hydraulic pressure (reproduced from [180]).

Yuk et al. [180] created a bi-layered hydrogel actuator which could respond to changes in the hydraulic pressure. The top layer hydrogel was shaped into several serial units which served as hydraulic chambers. The bottom layer was a pad made from a hydrogel stiffer than the top layer. Both top and bottom layers were constructed by tough alginate/PAAm IPN hydrogel, with the stiffness of the hydrogel that was modulated by the content and crosslinking density of alginate. When the two layers were combined, an elastomeric tubing was connected to the hydraulic chambers (Figure 14a). When water was pumped into the chambers, the actuator bent quickly in response to the increased hydraulic pressure. The actuator recovered to its original state when the water was removed (Figure 14b). Compared to hydrogel actuators which rely on the swelling driven by the diffusion of ions, the hydraulic actuator demonstrated a shorter responsive time, generated a higher actuation force, and remained transparent during actuation. This actuator may have potential in fabricating camouflaging robots in aqueous environments.

**Figure 15:**
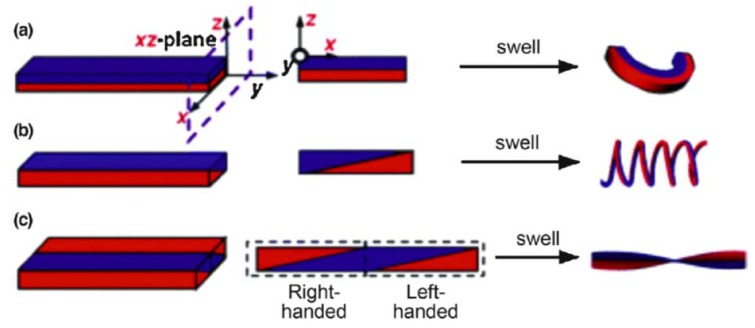
Schematic of (**a**) bending and (**b**) polypeptide-like twisting of bi-layered hydrogel. (**c**) Schematic of DNA-like twisting of tri-layered hydrogel. The blue and red parts represent hydrogels with different swelling ratios (reproduced from [181], copyright 2011 RSC).

In addition to bending, hydrogels can perform other types of deformation, such as twisting. In Jeong et al.’s work [10,181], by modifying a parallel bi-layered structure into a sloped structure, the swelling of the hydrogel can result in a polypeptide-type twisting deformation (Figure 15b). When the hydrogel is fabricated to be tri-layered with a sloped structure, it can twist into a DNA-like spiral geometry (Figure 15c).

## 6. Future Outlooks

Currently, one of the key challenges involves the need for a rapid and biocompatible in situ curing chemistry for preparing injectable tough hydrogels. The ability for the hydrogel to be delivered as a liquid that can solidify to adopt the new geometry of the tissue defect is critical for minimally-invasive delivery of the biomaterial for tissue repair and regeneration. Additionally, developing 3D printable precursor solutions that can be extruded and cure rapidly is a need. However, existing injectable tough hydrogels have been reported to be mechanically weaker when compared to a hydrogel fabricated using the traditional solvent casting approach [140]. Moreover, extensive UV irradiation time or curing time is often required to form hydrogels with elevated toughness, which is not practical [20]. In addition, there is a need to design tough hydrogels with a tunable degradation rate. Given that many of these biomaterials are designed for load-bearing applications, it is critical to understand how the mechanical property of tough hydrogels changes as it degrades while minimizing critical structural failure that may adversely affect the intended function of the hydrogel. Finally, the latest computer-aided design and manufacturing technologies, in combination with tough hydrogels may greatly enhance the ability of scientists to fabricate artificial tissues and medical devices with complex geometries and refined micro- to macro- structures [182]. Zhang et al. [183] have demonstrated an example of engineering a vascularized myocardium tissue for in vitro drug screening by integrating the 3D-printing of cell-laden IPN hydrogel, microfluids technique and stem cell technology. Such work exemplifies new opportunities for using tough hydrogel technology in the biomedical field. 

## 7. Summary

Tough hydrogels attempt to mimic both chemical and mechanical properties of native tissue for diverse biomedical applications. This work reviewed various reported strategies used to create tough hydrogels. These strategies involved the introduction of sacrificial bonds and viscous dissipation properties into the hydrogel network to maintain the network architecture during repeated large strain deformation. Tough hydrogels designed for biomedical applications, especially in the areas of tissue adhesive, tissue engineering, and soft actuator are introduced. These designs have the potential to open up new avenues to improve current clinical techniques, artificial tissue fabrications, and soft bioelectronics.
